# Compact all-optical precision-tunable narrowband hard Compton X-ray source

**DOI:** 10.1038/s41598-022-20283-8

**Published:** 2022-09-26

**Authors:** T. Brümmer, S. Bohlen, F. Grüner, J. Osterhoff, K. Põder

**Affiliations:** 1 Deutsches Elektronen-Synchrotron DESY, Notkestr. 85, 22607 Hamburg, Germany; 2 Universität Hamburg and Center for Free-Electron Laser Science, Luruper Chaussee 149, 22761 Hamburg, Germany

**Keywords:** Plasma-based accelerators, X-rays

## Abstract

Readily available bright X-ray beams with narrow bandwidth and tunable energy promise to unlock novel developments in a wide range of applications. Among emerging alternatives to large-scale and costly present-day radiation sources which severely restrict the availability of such beams, compact laser-plasma-accelerator-driven inverse Compton scattering sources show great potential. However, these sources are currently limited to tens of percent bandwidths, unacceptably large for many applications. Here, we show conceptually that using active plasma lenses to tailor the electron bunch-photon interaction, tunable X-ray and gamma beams with percent-level bandwidths can be produced. The central X-ray energy is tunable by varying the focusing strength of the lens, without changing electron bunch properties, allowing for precision-tuning the X-ray beam energy. This method is a key development towards laser-plasma-accelerator-driven narrowband, precision tunable femtosecond photon sources, enabling a paradigm shift and proliferation of compact X-ray applications.

## Introduction

Since the demonstration of X-ray imaging by Röntgen in 1895^1^, development of bright X-ray sources has allowed harnessing the power of X-rays in ever-evolving and broadening fields of research and applications, leading to enormous advances in topics as varied as advanced imaging^2–5^ and radiotherapy^6–9^ modalities as well as in crystallography applications^10–13^. Examples of medical breakthroughs relying on brilliant X-ray sources include K-edge subtraction (KES) imaging^14^, a potential alternative to digital subtraction angiography, as well as X-ray fluorescence imaging (XFI)^15^, enabling in-vivo pharmacokinetic studies. Such advanced diagnostic and treatment modalities place strict requirements on X-ray bandwidth: KES requires percent-level X-ray bandwidths with precision energy tunability to above and below a K-edge (80.7keV for gold), with smaller energy separations leading to lower dose^16^. Indeed many other applications, such as serial crystallography^10,12^, demand percent level bandwidths at tens of keVs. Additionally, femtosecond X-ray pulse duration and low jitter to short-pulse laser systems enable time-resolved pump-probe studies^11^ and the investigation of matter under extreme conditions^17^. For these and many other use cases^18,19^, a compact X-ray source of precision-tunable narrowband radiation could trigger disruptive progress and catalyse their adoption into applications of high societal relevance.

The final frequency $\omega _\mathrm {x}$ of a photon with initial frequency $\omega _\mathrm {L}$ Compton-scattered off an electron of energy $\gamma _e m_ec^2$, ignoring electron energy loss, is given by^20^

1 $$\begin{aligned} \omega _\mathrm {x} = \frac{2\gamma _e^2(1-\beta \cos \theta _I)}{1+\gamma _e^2\theta _O^2 + a^2/2 }\, \omega _\mathrm {L}, \end{aligned}$$

where $\theta _I$ and $\theta _O$ are the scattering and observation angles, respectively, $\beta =\sqrt{1-\gamma _e^{-2}}$ with electron Lorentz factor $\gamma _e$. $a=q_eA/m_ec$ is the normalised laser vector potential with peak value $a_0$, where *A* is the vector potential of the laser field, *c* is the speed of light and $q_e$ and $m_e$ are elementary charge and electron mass, respectively. For $\gamma _e \gg 1$, $\theta _I\simeq \pi$ and $a_0\ll 1$, the central frequency observed on the electron beam propagation axis is $\omega _\mathrm {x} \simeq 4\gamma _e^2 \omega _\mathrm {L}$. The axial bandwidth of the scattered X-ray beam (see “Methods”) arises predominantly from the electron bunch energy spread $\delta \gamma _e$ and divergence $\sigma _\theta$^21,22^, along with a permille to percent level contribution from laser bandwidth $\delta \omega _\text {L}$.

Generation of narrow bandwidth X-ray beams thus requires an electron bunch with very low energy spread and divergence, leading to narrowband inverse Compton-scattering (ICS) sources previously being demonstrated with conventional accelerators^23,24^. Quasi-monoenergetic electron spectra from laser plasma acceleration (LPA) have allowed for the demonstration of extremely compact and tunable all-optical ICS sources with peaked photon spectra^25–27^. However, with central energy and bandwidth of the X-ray beam directly derived from the electron bunch properties, milliradian divergence and $\gtrsim {10}\,{\%}$ relative energy spread results in demonstrated X-ray bandwidths of tens of percent^25–28^, unacceptably large for all applications discussed above. State-of-the-art LPAs providing 2–3% FWHM energy spreads^29,30^ would result in X-ray pulses with at least 4–6% FWHM bandwidth, too large for KES.

**Figure 1:**
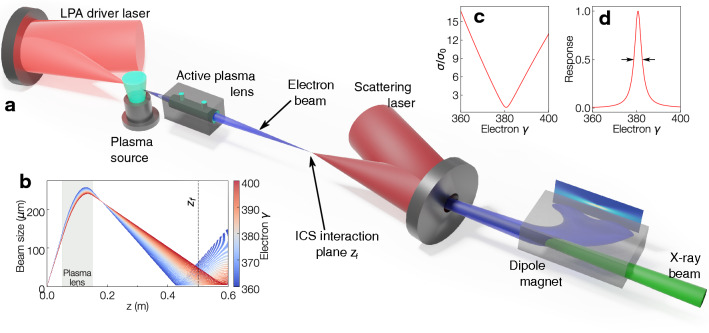
Schematic overview of the active plasma lens-tunable X-ray source. (**a**) A laser beam is focussed into a plasma source, generating an electron bunch. The electron bunch is captured and refocussed chromatically using an active plasma lens and interacts with a focussed scattering laser (potentially from a different, synchronised laser system) at a plane $z_f$, generating X-rays via ICS. The electron bunch is deflected with a dipole magnet, leaving the X-ray beam. (**b**) Trajectories of electrons with different energies being focussed by an active plasma lens, highlighting the chromaticity of the focussing. (**c**) The energy-dependence of RMS electron spot size at $z_f$. (**d**) The filtering response function Eq. (2) as a function of electron energy. The trajectories are calculated with lens current $I_{APL}={500}\,{\hbox {A}}$.

## Results

Here, we propose advanced control over the X-ray beam central energy and bandwidth by employing an active plasma lens (APL), a compact, high-strength radially symmetric focussing device^31–33^. The chromatic focussing of the APL allows tuning the central energy of the X-ray beam and reducing the effective energy spread and divergence of the electron bunch interacting with the scattering laser. Such advanced tailoring of the electron bunch allows LPA-driven X-ray beams with unprecedented percent-level X-ray bandwidths and precision energy tunability.

The schematic setup of the proposed precision-tunable ICS X-ray source is depicted in Fig. 1a. Electrons in a plasma wakefield-accelerated femtosecond-duration bunch are focussed by an APL to longitudinal positions dependent on their energy, as depicted in Fig. 1b. Figure 1c shows the variation of electron bunch RMS size $\sigma (\gamma _e)$ at the Compton interaction plane $z_f$. For interaction of a laser beam and electron bunch with gaussian profiles (see Supplementary Material S1), the response function $N(\gamma _e)$^34^, plotted in Fig. 1d, is given by

2 $$\begin{aligned} N(\gamma _e) \propto \frac{1}{ \left( \frac{2\sigma (\gamma _e)}{ w_0 }\right) ^ 2 + 1}, \end{aligned}$$

showing that only energy slices focussed to a spot size similar to that of the laser waist $w_0$ contribute significantly to the X-ray spectrum. The effective electron energy spread is given by the width of $N(\gamma _e)$ and can be much smaller than the bandwidth of the electron bunch. Varying the current flowing through the APL and thus its focussing strength results in electrons with different energies interacting with the scattering laser at $z_f$, thereby changing the X-ray beam energy.

**Figure 2:**
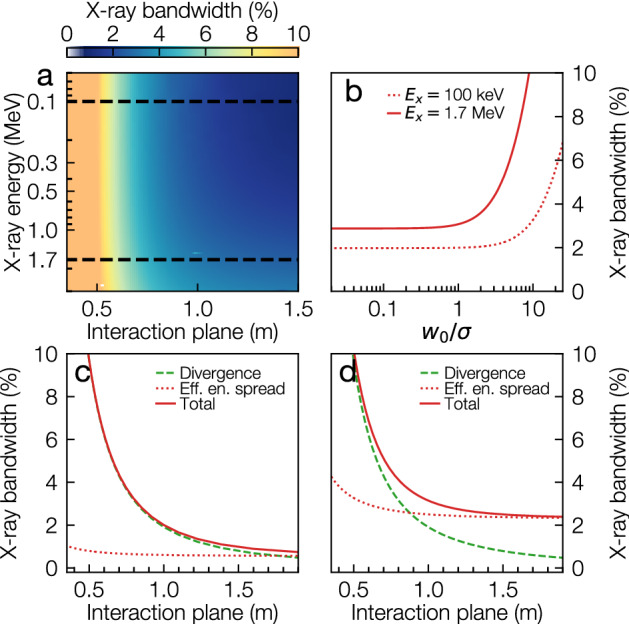
X-ray beam FWHM bandwidth arising from electron bunch properties. (**a**) On-axis X-ray bandwidth as a function of central energy and interaction plane for $w_0={10}\,{\upmu \hbox {m}}$. (**b**) The variation of X-ray bandwidth with laser-electron bunch overlap. (**c,d**) Variation of X-ray bandwidth as a function of interaction plane $z_f$ for X-ray energies of (**c**) ${100}\,{\hbox {ke}\,\hbox {V}}$ and (**d**) ${1.7}\,{\hbox {Me}\, \hbox {V}}$ with $w_0={10}\,\upmu \hbox {m}$. The dashed lines in panel (**a**) highlight the positions of the lineouts shown in panels (**c**) and (**d**). The electron bunch divergence-driven bandwidth component decreases rapidly with focus distance with the effective energy spread staying almost constant. The X-ray bandwidth calculations assume a ps-duration laser pulse length matched to the interaction volume.

The FWHM X-ray bandwidth contribution from electron bunch divergence and effective energy spread is presented in Fig. 2. The X-ray bandwidth shown in panel (a) decreases rapidly with increasing distance from the plasma lens and reaches a plateau, meaning moderate distances $z_f \approx {0.75}\,{\hbox {m}}$ are preferable to keep the system compact. In the plateau region, the relative bandwidth exhibits a weak dependence on the central X-ray energy, with a threefold increase from 1 % to 3.2 % between $E_x={100}\,{\hbox {ke}\,\hbox {V}}$ and $E_x={3}\,{\hbox {Me}\,\hbox {V}}$ at $z_f={1.5}\,{\hbox {m}}$. The X-ray bandwidth dependence on laser focal spot size is plotted in Fig. 2b, highlighting that the effective electron energy spread is minimized only if $w_0$ is of similar size or smaller than $\sigma$. In Fig. 2c the variation of X-ray bandwidth with the interaction plane is plotted for $E_x={100}\,{\hbox {ke}\,\hbox {V}}$, an energy suitable for gold-nanoparticle XFI, while in Fig. 2d this is plotted for $E_x={1.7}\,{\hbox {Me}\,\hbox {V}}$, the nuclear resonance line energy for $\mathrm {U}^{235}$. The X-ray bandwidth reduction at larger focal distances is driven by the reduction of the electron bunch divergence while bandwidth at large $z_f$ is dominated by the effective electron bandwidth at APL focus. For the X-ray energy range presented here, percent level bandwidths are attainable. This is a more than one order of magnitude improvement compared to previous results, where $\sim {50}\,{\%}$ bandwidths were measured^25,26^, enabling advanced applications such as KES.

**Figure 3:**
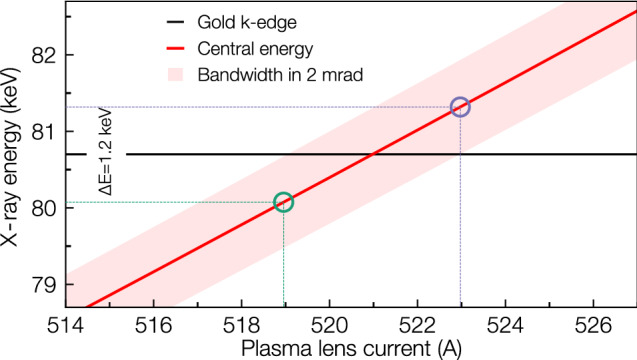
Precision tuning the X-ray beam energy with plasma lens current. The central Compton scattered X-ray energy (red line) and the FWHM divergence emitted into a 2 mrad cone full-angle are shown. A narrow bandwidth allows a 1.2 ke V energy separation around the gold k-edge (black line).

The precision energy tunability of the Compton source is presented in Fig. 3, showing the variation of X-ray energy and FWHM bandwidth with APL current for $r_\mathrm {APL}={2}\,{\hbox {mm}}$. The electron energy focused at the interaction plane varies linearly with APL current (see Supplementary Material S1), facilitating the tuning of the X-ray energy. Assuming an energy-independent normalised emittance $\varepsilon _N$, the variation of electron bunch spot size $\sigma$ with current is negligible, allowing for optimal laser-electron overlap $w_0/\sigma$ to be maintained. A percent-level X-ray bandwidth allows for an energy separation of only 1.2 ke V centred on the gold K-edge. This close separation, important for dose reduction^16^, is only possible due to the APL-tailoring in our source, as a 1.2 % X-ray bandwidth would otherwise require $\ll {0.6}\,{\%}$ electron bunch energy spread. Varying the APL current from 0.2kA to 1kA allows, in principle, generating X-ray beams with energy from few keV to 300 ke V without changes to the setup, a tuning range of nearly two orders of magnitude, given a broadband electron source.

Estimating the number of X-ray photons (see “Methods”) in a pencil beam with $2\theta _c={2}\,{\hbox {mrad}}$ at an X-ray energy of 80.7 ke V, with $\gamma _e=114$, effective energy spread $\delta \gamma _e/\gamma _e\simeq {0.3}\,{\%}$, a spectral density of $\mathrm {d}Q/\mathrm {d}\gamma _e ={10}\,\hbox {pC}/\hbox {Me}\,\hbox {V}$^35^, laser peak normalised vector potential $a_0=0.3$, $w_0={15.6}\,{\upmu \hbox {m}}$ and $\tau _L={1}\,{\hbox {ps}}$ results in 1.2 x 10 photons in an X-ray bandwidth of 1.5 % for the configuration used for Fig. 3. This corresponds to a peak brightness^36^ of $1.4\times 10^{21}\,{\hbox {photons}/ \hbox {s}/\,\hbox {mm}^{2}/\hbox {mrad}^{2}/ ({0.1}\% \hbox {BW})}$ assuming pulse duration $\tau ={10}\,{\hbox {fs}}$. The configuration can be changed by optimising the electron focus and laser spot size and duration to maximise yield at a particular energy^37^ at the expense of tuning range, resulting in potentially an order of magnitude increase of photon number at the target energy.

## Discussion

In addition to allowing spectral tailoring and tuning, introduction of the APL in the scattering setup has many stability benefits. As the electron source is imaged and magnified at the ICS interaction point, spectral fluctuations arising from electron bunch pointing variations are reduced by the magnification factor. X-ray bandwidth changes arising from potential LPA electron source spectrum fluctuations are also lowered as the energy of electrons interacting with the laser is determined by the APL focussing, dominated by the reproducibility of the current pulse which is on the few permille level^33^. Importantly, the use of APLs instead of quadrupole magnet doublet and triplet systems offers significant advantages. The radially symmetric focussing is performed with a single element, requiring tuning of only one parameter, while focussing strengths of the order of kT/m allow construction of a narrowband multi-MeV photon source on a metre scale. Finally, our method of tailoring the electron beam properties allows *generating* narrowband X- or $\gamma$-ray radiation in a highly compact setup, offering many benefits compared to filtering the bandwidth of a wideband photon beam post-ICS interaction, such as further simplification of the experimental setup, simplified operation procedure and reduced radiological shielding.

It is instructive to compare the performance of the compact all-optical X-ray source concept presented in this work with presently available alternatives, particularly in the high energy X-ray regime. Currently available state-of-the-art compact light sources such as liquid-metal-jet microfocus tubes^38^ can produce a flux of ${2\times 10^{6}}\,{\hbox {photons}/\hbox {s}}$ into a cone of $2\theta _c={2}\,{\hbox {mrad}}$ and bandwidth of 1.2 ke V at a photon energy of 80 ke V; flux can be considerably higher at lower energies, near k-edges. This is orders of magnitude lower than our work operating at 1 kHz, further, the CW nature of microfocus tubes rules out any time-resolved studies on a timescale faster than few tens of milliseconds. Proposed upgrades to the RF-driven ICS source Lyncean CLS ^39^ allow operation also at 80 ke V energies, delivering $\gtrsim {6\times 10^{11}}\,{\hbox {photons}/\hbox {s}}$ into $2\theta _c={2}\,{\hbox {mrad}}$ cone with percent level bandwidth. The pulse duration of these machines is tens of picoseconds, allowing no access to femtosecond phenomena, while the design of the machine does not allow e.g. rotating gantry-style X-ray beam delivery. Some synchrotrons can operate at energies $\sim {80}\,{\hbox {ke}\,\hbox {V}}$^40–42^ and beyond up to $\simeq {200}\,{\hbox {ke}\,\hbox {V}}$, providing flux of the order $1\times 10^{11}-1\times 10^{12}\,{\hbox {photons}/\hbox {s}}$ into permille level bandwidths, however these are very large scale machines with typically picosecond pulse durations, also not able to access ultrafast timescales. As the geometric emittance of the electron beam in a free-electron laser (FEL) needs to be of the order of the generated radiation wavelength, currently no machines operate beyond $\sim {25}\,{\hbox {ke}\,\hbox {V}}$; further injector and accelerator technology upgrades are required to push FELs to energies beyond that. Our method allows also scaling to MeV-level photon energies while still maintaining a compact footprint, something that is out of reach for the sources relying on conventional accelerator technology outlined above.

The results presented in Fig. 3 neglect the bandwidth contribution of the scattering laser pulse. This approximation is justified for laser systems with transform-limited pulse duration $\tau _L$ of picosecond order; the synchronisation of these lasers to the LPA-driver can be on the level of ${100}\,{\hbox {fs}}\ll \tau _L$^21^. Scattering laser pulses split from LPA-driver can be carefully chirped to reduce the effective bandwidth of the laser pulse in the interaction volume^37,43^, resulting in negligible contributions to X-ray bandwidth.

In conclusion, we demonstrate a novel concept for a low-divergence Compton scattering X-ray source providing precision tuning at X-ray energies from keV to MeV and a narrow bandwidth. The all-optical architecture of our source enables a vast reduction of the machine size compared to RF-accelerator-based machines, potentially allowing for a portable high-brightness hard X- or $\gamma$-ray source. The ongoing development of high power kilohertz repetition rate laser systems^44–46^ can lead to photon fluxes $>10^8$ photons/s in percent-level bandwidths in a few-mrad pencil beam. In addition to various imaging applications, a light source with the demonstrated tuning range could open the possibility for a single compact device to be used for both diagnostics^37^ and subsequent precision radiation therapy^9^. The source demonstrated here will dramatically expand access to high brightness, narrow-band, tunable photon sources for a wide range of scientific and medical applications while the femtosecond duration and inherent optical synchronisation will enable many more advanced pump-probe experiments.

## Methods

### Plasma lenses

Active plasma lenses^31–33^ rely on the azimuthal magnetic field generated by a current $I_{APL}$ flowing collinearly with an electron bunch. Typically operated in a discharge plasma, the current density can in the ideal case be modelled as being radially uniform leading to linear magnetic field gradients given by $\partial B_\phi /\partial r = \mu _0 I_{APL}/(2\pi r_{APL}^2)$, with $r_{APL}$ being the radius of the plasma lens. The focussing strength for electrons of energy $\gamma _e m_ec^2$ is given by $k=q_e/(\gamma _e m_e c)\partial B_\phi /\partial r$. For all calculations in this manuscript the plasma lens starts at $z_u={5}\,{\hbox {cm}}$ and is $L_\mathrm {APL}={10}\,{\hbox {cm}}$ long, its radius is $r_\mathrm {APL}={1}\,{\hbox {mm}}$ unless specified otherwise.

### Electron bunch focussing

Beam transport matrices are used to calculate the Courant-Snyder parameters $\alpha$, $\beta$, $\gamma$^47^ using

3 $$\begin{aligned} \begin{pmatrix} \beta \\ \alpha \\ \gamma \end{pmatrix} = \begin{pmatrix} C^2 &{} -2SC &{} S^2 \\ -CC'&{} S'C-SC' &{} -SS' \\ C'^2 &{} -2S'C' &{} S'^2 \end{pmatrix} \begin{pmatrix} \beta _i\\ \alpha _i \\ \gamma _i \end{pmatrix} \end{aligned}$$

with $C=\cos \phi$, $S=\sin \phi /\sqrt{k}$, $C'=-\sqrt{k}\sin \phi$, $S'=\cos \phi$ and $\phi =L\sqrt{k}$. For drift space, $k_\mathrm {drift}=0$ and $L_\mathrm {drift}\equiv \Delta z$ and for the plasma lens, $L\equiv L_\mathrm {APL}$ and $k=k_\mathrm {APL}$. The focal plane can be calculated by setting $\alpha =0$, see Supplementary Information S1. At a given focal plane $z_f$, the focussed energy varies linearly with plasma lens current $\gamma _e \propto I_\mathrm {APL}$. For all the simulations and beam transport calculations, an electron bunch with $\epsilon _N={1}\,\upmu \hbox {m}$ and $\sigma ={1}\,\upmu \hbox {m}$ at $z=0$ is tracked.

### X-Ray bandwidth and photon number

The FWHM bandwidth theory by Rykovanov et al.^21^ was applied to calculate the X-ray bandwidth. In the linear regime $a_0\ll 1$ it is governed by the energy spread $\delta \gamma _e/\gamma _e$ and FWHM divergence $\sigma _\theta$ of the electron bunch, the FWHM laser bandwidth $\delta \omega _\text {L}/\omega _\text {L}$ and the collimation angle $\theta _c$:

4 $$\begin{aligned} \frac{\Delta \omega _x}{\omega _x} =\sqrt{\left( \frac{2\delta \gamma _e}{\gamma _e}\right) ^2 +\bigg (\frac{\gamma _e^2\sigma _{\theta }^2}{4}\bigg )^2 +\bigg (\frac{\delta \omega _\text {L}}{\omega _\text {L}}\bigg )^2+ \kappa ^2} , \end{aligned}$$

with $\kappa = \gamma _e^2\theta _c^2/(1+\gamma _e^2\theta _c^2)$. The number of X-ray photons within a bandwidth $\kappa$ arising from an opening angle $\theta _c$ can be estimated by^21^

5 $$\begin{aligned} N_{\text {L}}=\kappa \left( \kappa ^2 - \frac{3}{2}\kappa + \frac{3}{2}\right) \frac{\alpha _f}{3}\frac{Q_\text {eff}}{q_e} \, \tau _L \omega _\text {L} a_0^2, \end{aligned}$$

where $\alpha _f\approx 1/137$ is the fine-structure constant, $a_0$ is the peak normalised vector potential and $\tau _L$ is the intensity FWHM duration of the laser, and

6 $$\begin{aligned} Q_\mathrm {eff} \sim \int _{\gamma _e - \delta \gamma _e}^{\gamma _e + \delta \gamma _e}{\frac{\mathrm {d}Q}{\mathrm {d}\gamma _e}\,\mathrm {d}\gamma _e} \end{aligned}$$

is the effective number of electrons interacting with the laser.

### Simulations of microfocus X-ray source

The Monte-Carlo code penelope^48^ was used to simulate the bremsstrahlung emitted by a liquid-jet microfocus X-ray tube. A homogenous anode alloy containing 47 % Ga, 37 % In and 16 % Sn was used as the target^49^. The electron beam focus size was ${10}\,{\upmu \hbox {m}}$, an anode-target angle of ${11}^\circ$ and a take-off angle of ${25}^\circ$ were used, all other parameters were the default values in penelope. A voltage of 160 ke V and a state-of-the-art current of 6.25 mA were assumed^49^.

## Supplementary Information

Supplementary Information.

## Data Availability

The datasets used and analysed during the current study are available from the corresponding author on reasonable request.
