# Supplementary material for: Compact all-optical precision-tunable narrowband hard Compton X-ray source
Source: Sci Rep. 2022 Sep 26;12:16017. doi: 10.1038/s41598-022-20283-8 (PMC9512799; doi:10.1038/s41598-022-20283-8)
Supplement: Supplementary file 1 — Supplementary Information. [file 41598_2022_20283_MOESM1_ESM.pdf]

Supplementary: Compact all-optical precision-tunable narrowband hard Compton X-ray source

T. Brümmer,[1] S.Bohlen,[1] F. Grüner,[2] J. Osterhoff,[1] and K. Põder[1,*]

[1] *Deutsches Elektronen-Synchrotron DESY, Notkestr. 85, 22607 Hamburg, Germany*
[2] *Universität Hamburg and Center for Free-Electron Laser Science, Luruper Chaussee 149, 22761 Hamburg, Germany*

(Dated: September 7, 2022)

I. PERMANENT-MAGNET QUADRUPOLES VS. ACTIVE PLASMA LENSES

An active plasma lens (APL) is an attractive novel focussing device. An advantage over systems employing multiple permanent-magnet quadrupoles (PMQs) lies in very strong and compact, single-element symmetric focussing. This is highlighted in Fig 1, showing the focal spot size and beam divergence at focus for an APL and a PMQ doublet. Here, the APL is $L = 10\,\mathrm{cm}$ long and positioned at $z_0 = 5\,\mathrm{cm}$ from the beam source at $z = 0$ [1]; the PMQs in the doublet have a field gradient of $\partial B/\partial r = 500\,\mathrm{T\,m^{-1}}$ and lengths of $L_1 = 17\,\mathrm{mm}$ and $L_2 = 15\,\mathrm{mm}$. As can be seen, the focal spot in the APL case is not only symmetric, but also stays constant in size as electrons with different energies are focussed whereas the physical relocation of permanent magnet PMQs results in variation of spot size and divergence. Further, while for the PMQ doublet the variation is extremely different in the two transverse planes, the divergence at focus is monotonically decreasing in the APL case. These benefits add to the lack of any moveable parts in the APL case.

The use of more complex conventional focussing systems, such as PMQ triplets or electromagnetic quadropole magnets, can lead to spot size and divergence variation more similar to the APL case. However, a focussing PMQ triplet adds even more complexity and moving parts to the setup while electromagnetic quadropole magnets are bulky, negating the opportunity for building a compact light source based on the concept in [1].

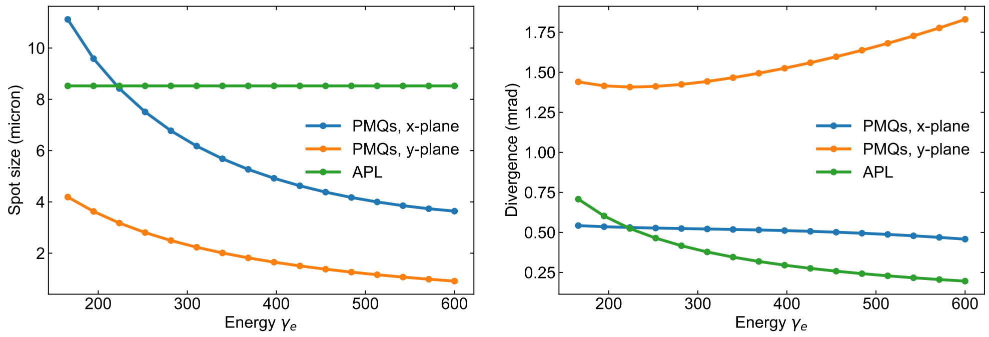

FIG. 1: Comparison of the focal spot size (a) and beam divergence at focus (b) as a function of energy for an active plasma lens and a permanent magnet quadrupole doublet focussing the beam to $z = 1\,\mathrm{m}$.

II. CHROMATIC APL FOCUSING AND INVERSE COMPTON SCATTERING

A. Tunability through APL current

Changing the plasma lens current I_{APL} varies the focusing strength $k \propto I_{\mathrm{APL}}$ of the APL. Thus, at a given interaction plane with the Inverse Compton Scattering (ICS) laser, the focused electron energy slice can be tuned via I_{APL}.

Calculation of the full beam transport, i.e. the Courant-Snyder Parameters (CSPs), from the accelerator output to the ICS interaction relies on the free drift matrix M_{FD} and the beam transport matrix through the active plasma lens M_{APL} given by

*Electronic address: kristjan.poder@desy.de

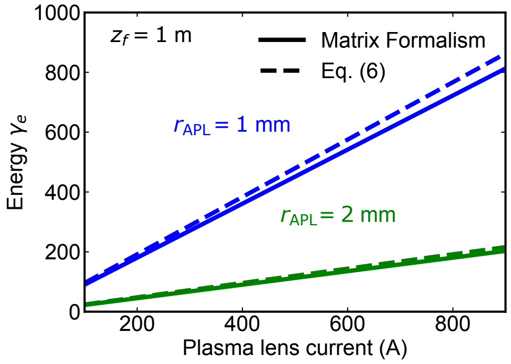

FIG. 2: Focused electron energy γ_e as a function of the plasma lens current, for the full matrix calculation (solid lines) and the approximation in Eq. (6) (dashed lines).

$$M_{\mathrm{FD}} = \begin{pmatrix} 1 & -2\Delta z & \Delta z^2 \\ 0 & 1 & -\Delta z \\ 0 & 0 & 1 \end{pmatrix} \tag{1}$$

$$M_{\mathrm{APL}} = \begin{pmatrix} C^2 & -2SC & S^2 \\ -CC' & S'C - SC' & -SS' \\ C'^2 & -2S'C' & S'^2 \end{pmatrix} \tag{2}$$

with drift length Δz, $C = \cos\phi$, $S = \sin\phi/\sqrt{k}$, $C' = -\sqrt{k}\sin\phi$, $S' = \cos\phi$, $\phi = L\sqrt{k}$, APL length L and

$$k = \frac{q_e}{\gamma_e m_e c}\frac{\partial B_\phi}{\partial r} \tag{3}$$

is the focussing strength of the APL.

With z_u the distance from the electron source to the APL and α, γ the CSPs at the APL exit, the distance to focus after the APL z_v is given by

$$z_v = \frac{\alpha}{\gamma} \approx \frac{\sqrt{k} z_u \cos\phi + \sin\phi}{-\sqrt{k}\cos\phi + k z_u \sin\phi} \approx \frac{\sqrt{k} z_u (1 - \frac{\phi^2}{2}) + (\phi - \frac{\phi^3}{6})}{-\sqrt{k}(1 - \frac{\phi^2}{2}) + k z_u (\phi - \frac{\phi^3}{6})} \tag{4}$$

for sufficiently small ϕ.

Assuming linear focussing fields with a field gradient $\partial B_\phi/\partial r = \mu_0 I_{\mathrm{APL}}/(2\pi r_{APL}^2)$, with r_{APL} being the radius of the plasma lens, Eq.(4) can be solved for γ_e, yielding the focused electron energy at the distance z_v from the APL exit:

$$\gamma_e(I_{\mathrm{APL}}) = \frac{K I_{\mathrm{APL}} L}{12 r_{APL}^2 z_f} \times \Big[L^2 + 3L z_u + 3 z_v (L + 2 z_u) \tag{5}$$

$$+ \sqrt{L^2(L + 3z_u)^2 + 6 z_v L (L^2 + L z_u + 2 z_u^2) + 3 z_v^2 (3L^2 + 4L z_u + 12 z_u^2)} \Big]$$

$$\approx \frac{KL}{12 r_{APL}^2 z_f} I_{\mathrm{APL}}, \tag{6}$$

where $z_f = z_u + L + z_v$ is the interaction plane and $K = q\mu_0/(2\pi m_e c)$.

The electron energy focused at a fixed interaction plane thus depends linearly on the APL current I_{APL} and the X-ray energy $E_x \propto I_{\mathrm{APL}}^2$. The tuning range can be adjusted via the APL length L, radius r_{APL}, position z_u and the interaction plane z_f. Fig. 2 shows the good agreement of Eq. (6) with the matrix formalism for lenses of $r_{APL} = 1$ mm and $r_{APL} = 2$ mm.

B. Derivation of the effective energy spread

In ICS, photon emission depends on the overlap of electrons and laser pulse [2, 3]. The smaller the mean bunch size compared to the laser waist, the larger the mean laser field acting on the electrons, and thus the

emitted photon number. Due to chromatic focusing of the APL, each energy slice (γ_e) is focused at a different position $f(\gamma_e)$. Bunch slices of energies deviating from the target electron energy are focused before or after the laser focus. Consequently, they have a larger size in the interaction center and emit less photons. The *effective energy spread* is defined as the width of $N_x(\gamma_e)$, the number of emitted photons by an electron of energy γ_e.

The number of photons emitted in an ICS interaction of a laser with pulse duration τ_L and a bunch with charge Q is

$$N_x \propto \tau_L a_{\text{eff}}^2 Q \tag{7}$$

with the mean effective laser strength parameter $a_{0,\text{eff}}$. For sufficiently small Thomson interaction lengths compared to the electron bunch beta function the bunch size stays approximately constant during the interaction.

$$L_{\text{int}} = c(\tau_L + \tau_b)/2 \approx c\tau_L/2$$

for negligible bunch length $c\tau_b \ll c\tau_L$. This is a valid approximation, assuming typical parameters as in the manuscript [1]. Consequently, longitudinal effects can be neglected and the effective laser strength and photon number can be determined via the two-dimensional overlap on the laser focus. For Gaussian transverse laser-intensity and electron-bunch profiles, and $I \propto a^2$:

$$a^2(x,y) = a_0^2 \exp\left(-2\frac{x^2+y^2}{w_0^2}\right) \tag{8}$$

$$N_e(x,y) = N_{e,0} \exp\left(-\frac{x^2+y^2}{2\sigma_e^2}\right) \tag{9}$$

Weighting the intensity distribution with the spatial electron distribution gives the effective laser strength parameter

$$a_{\text{eff}} = \sqrt{\frac{\int_{-\infty}^{\infty}\int_{-\infty}^{\infty} a^2(x,y)N_e(x,y)\mathrm{d}x\mathrm{d}y}{\int_{-\infty}^{\infty}\int_{-\infty}^{\infty} N_e(x,y)\mathrm{d}x\mathrm{d}y}} \tag{10}$$

$$= \frac{a_0 w_0}{\sqrt{4\sigma_e^2 + w_0^2}} \tag{11}$$

So that

$$N_{\text{eff}}(\gamma_e) \propto \frac{a_0^2}{4\sigma_e^2(\gamma_e)/w_0^2 + 1}. \tag{12}$$

With the beta function β_0 in the slice focus $f(\gamma_e)$ and the drift length Δz between the focal point for a given electron energy γ_e and the target-energy focus ($f(\gamma_{e,t}) = f_L$), the bunch size is:

$$\sigma_e(\gamma_e, f_L) = \sqrt{\frac{\varepsilon_n}{\gamma_e}\left[\beta_0(\gamma_e) + \frac{(\Delta z(\gamma_e))^2}{\beta_0(\gamma_e)}\right]} \tag{13}$$

For sufficiently large γ_e and small $\Delta\gamma_e$, the square of the electron slice size is approximately symmetric and of the form $\sigma_e^2(\gamma_e) \propto \gamma_e^2 + x$, with variable x. Thus, $N_{\text{eff}}(\gamma_e)$ can be described by a Lorentzian and the FWHM width is the effective electron energy spread.

C. Tunability and bandwidth of APL-tunable X-ray source

A shown above, the current variation in the APL accommodates energy tuning of the X-ray beam. The current dependence of central X-ray energy and bandwidth is plotted in Fig. 3a for $r_{APL} = 1\,\text{mm}$, $z_0 = 5\,\text{cm}$ and $z_f = 1\,\text{m}$ for a beam with energy-independent normalised emittance $\epsilon_0 = 1\,\mu\text{m}$. The scattered X-ray energy varies from 0.05 MeV to 4.8 MeV, a tuning range of nearly 2 orders of magnitude. The relative X-ray bandwidth emitted into a 1 mrad cone increases by a factor of 6 over this tuning range. The black line in Fig. 3a is the inherent ICS bandwidth $\kappa = \gamma^2\theta_c^2/(1+\gamma^2\theta_c^2)$ due to the $\theta_c = 1\,\text{mrad}$ collimation angle, highlighting that the bandwidth is limited by the observation angle and other contributions to the bandwidth have been effectively reduced.

For a given plasma lens current range, the X-ray tuning range can be shifted to lower energies by increasing the APL radius and thus reducing the APL strength $k \propto r_{APL}^{-2}$. As an example, in Fig. 3b the X-ray beam properties are plotted for a plasma lens with radius $r_{APL} = 2\,\text{mm}$. Without any other changes, the tuning

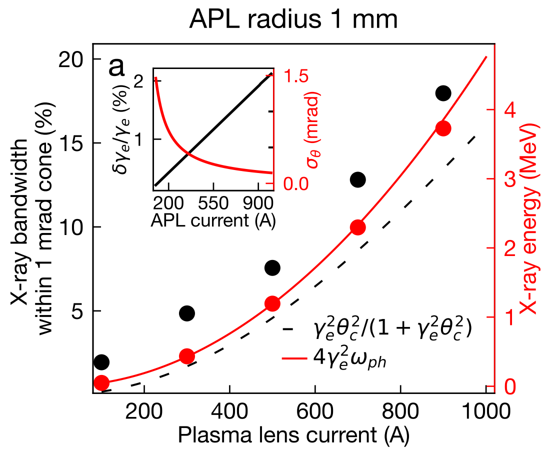

FIG. 3: Tuning the Compton X-ray beam properties with plasma lens current. The central Compton scattered beam energy and its FWHM bandwidth within a 1 mrad cone angle for plasma lens radius of **a** 1 mm and **b** 2 mm. The data points depict results from numerical simulations using a laser with $\tau_L = 1\,\mathrm{ps}$, $a_0 = 0.1$ and $w_0 = 2\sigma$. The insets show the variation of the electron bunch divergence and effective energy spread at the interaction plane with current. The red lines depict the $4\gamma_e^2$ scaling while the black lines show the bandwidth arising from a finite observation cone $\theta_c = 1\,\mathrm{mrad}$.

range is now from 3 keV to 300 keV, with the bandwidth increasing by a factor of 2.5, mainly due to a higher divergence. As the synchrotron angle $1/\gamma_e \gg 1\,\mathrm{mrad}$, the bandwidth is dominated by contributions from the electron bunch properties and not the observation cone.

The particle tracking code ASTRA [4] was used to calculate the electron trajectories taking into account space charge forces. An electron bunch, represented by 5×10^3 macroparticles, is propagated through the linear radial magnetic field of the plasma lens, and, in the interaction plane, through the time-varying electromagnetic fields of the bi-Gaussian (time and space) scattering laser. The classical radiation code CLARA2 [5] is employed to determine the photon angular spectrum. CLARA2 employs Lienard-Wiechert potentials to calculate the radiation emission on the basis of the macroparticle trajectories.

D. Oscillations in the capillary - multiple energies focused

For sufficient APL length L and focusing strength, low-energy electrons can be "focused" within the capillary, i.e. the spot size of that energy slice "oscillates". As a consequence, electrons of different γ_e focus to the same plane (see also Fig 2). This is illustrated in Fig. 4. This effect is negligible on the X-ray spectrum due to various reasons.

Firstly, low γ_e electrons obtain a larger divergence at given focusing strength k of the lens, and emit radiation into a larger synchrotron angle $\theta_s \sim \gamma_e^{-1}$ than high γ_e electrons, reducing their photon emission into the observation cone. Secondly, the longitudinal separation $\Delta f/\Delta\gamma_e$ is larger, reducing $\Delta Q/\Delta z$, i.e. the effective number of electrons interacting with the laser. Thirdly, as shown in the example in Fig. 4, the X-ray energies are orders of magnitude apart ($E_X(\gamma = 44) \sim 12\,\mathrm{keV}$, $E_X(\gamma = 443) \sim 1.2\,\mathrm{MeV}$), easily separated via appropriate filtering. For cases such as illustrated in Fig 4, a large fraction of electrons of low γ_e will not be focussed to z_f by the APL as they will leave the focussing fields within the APL when their excursion exceeds the lens radius. Finally, the low γ_e contribution to the X-ray spectrum will be determined and is thus controllable by the number of electrons in that spectral range.

[1] T. Brümmer, S. Bohlen, F. Grüner, J. Osterhoff, and K. Põder, submitted (2022).
[2] J. M. Krämer et al., Sci. Rep. **8**, 1 (2018).
[3] T. Brümmer, A. Debus, R. Pausch, J. Osterhoff, and F. Grüner, PRAB **23**, 031601 (2020).
[4] Using ASTRA, `https://www.desy.de/~mpyflo/`, accessed: 2022-09-06.
[5] R. Pausch, A. Debus, R. Widera, K. Steiniger, A. Huebl, H. Burau, M. Bussmann, and U. Schramm, Nucl. Instrum. Methods Phys. Res. A **740**, 250 (2014).

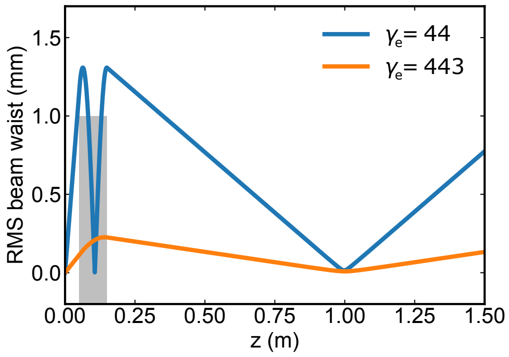

FIG. 4: Oscillation of lower γ_e electrons in the APL results in focussing to the same plane as electrons with higher energy.
